# Supplementary material for: Role and mechanism of NCAPD3 in promoting malignant behaviors in gastric cancer
Source: Front Pharmacol. 2024 Apr 22;15:1341039. doi: 10.3389/fphar.2024.1341039 (PMC11070777; doi:10.3389/fphar.2024.1341039)
Supplement: Supplementary file 11 [file DataSheet2.ZIP › GSEA/Oncogenic signature/my_analysis.Gsea.1599461962066/KRAS.600_UP.V1_UP.html]

Details for gene set KRAS.600\_UP.V1\_UP[GSEA]

|  || Dataset | filtered\_dataset.sample\_info.cls#WT\_versus\_NCAPD3\_MUT |
| Phenotype | sample\_info.cls#WT\_versus\_NCAPD3\_MUT |
| Upregulated in class | NCAPD3\_MUT |
| GeneSet | KRAS.600\_UP.V1\_UP |
| Enrichment Score (ES) | -0.51517445 |
| Normalized Enrichment Score (NES) | -2.155422 |
| Nominal p-value | 0.0 |
| FDR q-value | 0.010777657 |
| FWER p-Value | 0.04 |
Table: GSEA Results Summary

  

Fig 1: Enrichment plot: KRAS.600\_UP.V1\_UP      
 Profile of the Running ES Score & Positions of GeneSet Members on the Rank Ordered List

  

| SYMBOL | TITLE | RANK IN GENE LIST | RANK METRIC SCORE | RUNNING ES | CORE ENRICHMENT || 1 | 7052 | TGM2 | 17 | 1.045 | 0.0813 | No |
| 2 | 2921 | CXCL3 | 57 | 0.884 | 0.1325 | No |
| 3 | 7099 | TLR4 | 541 | 0.451 | -0.1737 | No |
| 4 | 3673 | ITGA2 | 858 | -0.264 | -0.3767 | No |
| 5 | 10123 | ARL4C | 957 | -0.377 | -0.4133 | No |
| 6 | 6795 | AURKC | 1100 | -0.477 | -0.4725 | Yes |
| 7 | 2012 | EMP1 | 1119 | -0.494 | -0.4412 | Yes |
| 8 | 3706 | ITPKA | 1150 | -0.514 | -0.4167 | Yes |
| 9 | 2119 | ETV5 | 1176 | -0.545 | -0.3859 | Yes |
| 10 | 6690 | SPINK1 | 1200 | -0.577 | -0.3508 | Yes |
| 11 | 80183 | KIAA0226L | 1208 | -0.585 | -0.3034 | Yes |
| 12 | 23143 | LRCH1 | 1209 | -0.585 | -0.2511 | Yes |
| 13 | 1358 | CPA2 | 1263 | -0.648 | -0.2311 | Yes |
| 14 | 1839 | HBEGF | 1328 | -0.751 | -0.2098 | Yes |
| 15 | 55521 | TRIM36 | 1337 | -0.774 | -0.1463 | Yes |
| 16 | 9966 | TNFSF15 | 1376 | -0.900 | -0.0930 | Yes |
| 17 | 4312 | MMP1 | 1407 | -1.304 | 0.0022 | Yes |
Table: GSEA details [plain text format]

  

Fig 2: KRAS.600\_UP.V1\_UP      
 Blue-Pink O' Gram in the Space of the Analyzed GeneSet

  

Fig 3: KRAS.600\_UP.V1\_UP: Random ES distribution      
 Gene set null distribution of ES for **KRAS.600\_UP.V1\_UP**
